# The history of liver surgery: Achievements over the past 50 years

**DOI:** 10.1002/ags3.12322

**Published:** 2020-02-26

**Authors:** Norihiro Kokudo, Nobuyuki Takemura, Kyoji Ito, Fuminori Mihara

**Affiliations:** ^1^ Department of Surgery National Center for Global Health and Medicine Tokyo Japan

## Abstract

We reviewed the progress made in the field of liver surgery over the past 50 years. The widespread use and improved outcomes of the hepatectomy were, primarily, due to pioneer surgeons who were responsible for technological advances and rapid improvements in the safety of the procedure in the last century. These advances included the hepatic functional evaluation used to determine the safety limit of liver resections, the introduction of intraoperative ultrasonography, and the development of innovative techniques such as portal vein embolization to increase the remnant liver volume. Cadaveric liver transplantation has been attempted since 1963. However, the clinical outcomes only began improving and becoming acceptable in the 1970s‐1980s due to refinements in technology and the development of new immunosuppressants. Partial liver transplantation from living donors, which was first attempted in 1988, required further technological innovation and sophisticated perioperative management plans. Moreover, these developments allowed for further overall improvements to take place in the field of liver surgery. Since the turn of the century, advances in computation and imaging technology have made it possible for safer and more elaborate surgeries to be performed. In Japan, preoperative 3‐dimensional simulation technology has been covered by health insurance since 2012 and is now widely used. An urgent need for real‐time navigation tools will develop in the future. Indocyanine green (ICG) fluorescence imaging was first used in 2007 and has led to the creation of a new surgical concept known as fluorescence navigation surgery. Laparoscopic surgery and robotic surgery have solved the issue of large incisions, which used to be a major drawback of open liver surgery; however, further improvements are required in order to achieve the level of safety and accuracy observed during open liver resection when performing all minimally invasive procedures. In the near future, liver surgery will become more precise and less invasive due to substantial progress including the development of navigation surgery, cancer imaging, and minimally invasive surgery. This overview of the history of liver surgery over the past 50 years may provide useful insights for further innovation in the next 50 years.

## 1 INTRODUCTION

Tremendous progress has been made in the field of liver surgery over the past 50 years. The safety and accuracy of performing liver resections have been improved dramatically, making it a current daily practice in many centers for the treatment of various liver diseases. Liver transplantation, the sole treatment option for patients with end‐stage liver disease, has also become a widely used, accessible surgical treatment option with acceptable surgical outcomes. Herein, we present an overview of the history of liver surgery over the last 50 years, highlighting the major pioneering events that took place, in order to provide insights which are useful for further innovation over the next 50 years.

## 2 PREHISTORY OF LIVER SURGERY

After the establishment of basic surgical techniques such as anesthesia and infection control in the late 19th century, many abdominal surgeries became feasible. In 1881, Billroth successfully performed a gastrectomy. In 1882, Langenbuch succeeded in performing a cholecystectomy, and later, in 1886, the world's so‐called first partial hepatectomy was attempted by Lius on a 67‐year‐old female patient with a hepatic adenoma. A hepatic tumor the size of a child's head was reportedly cauterized and resected using Paquelin's cautery; however, the patient died 6 hours after surgery due to postoperative hemorrhage. This complication is expected when considering the lack of availability of electrosurgical knives and the use of Paquelin's thermocautery, which consisted of burning flammable liquids such as benzene at a high temperature. Later, the hepatectomy was sporadically attempted, including the resection of metastatic liver cancers by Bruns (in the year 1888) and the resection of hemangiomas by von Eiselberg (in the year 1893). In 1889, Keen reported three cases of hepatectomies that he had experienced and 73 cases from the scientific literature, all of which consisted of the excision of a wedge‐shaped portion of the liver.^1^

In a report published in 1908, Doctor Pringle, a surgeon from Glasgow, performed open abdominal surgery on four patients with liver injuries and controlled the bleeding by obstructing the portal vein and hepatic artery, with one patient surviving as a result.^2^ In animal experiments, Pringle had also previously been successful in performing a unilateral hepatic lobectomy under hepatic inflow occlusion. Pringle's method is now widely used to reduce blood loss during liver transections.

The first report of the successful resection of the right hepatic lobe is said to have been performed by Wendel in 1911. The patient was a 44‐year‐old housewife who underwent extended right lobe resection for hepatocellular carcinoma (HCC); the resected specimen weighed 940 g, and the patient survived for 9 years. However, the details of the procedure are unclear, and although the right hepatic artery and hepatic duct were ligated and resected, the portal vein was not treated individually due to fear of thrombus formation.

On 16 October 1951, a French team led by Lortat‐Jacob performed a right hepatic lobectomy with systematic dissection of the hepatic hilum and, in 1952, they published a report on this case in French. The impressive graphic representation of the dissection of the hepatic hilum in their report later became internationally recognized.^3^ However, in Professor Foster's book, Solid liver tumor, published in 1977, he acknowledged that the first known case may have been a right hepatic lobectomy (with dissection of the hepatic hilum) reported by Honjo et al, in an English journal, that was accepted in 1953. Honjo et al^4^ succeeded in performing a right lobectomy with systematic dissection of the hepatic hilum on 7 March 1949, but published their case report in Japanese in 1950 and in English in 1955. They certainly deserve credit for succeeding in the world's first right hepatic lobectomy. Lumbar anesthesia was used, and Ringer's solution was administered by intravenous drip infusion. Honjo et al accomplished the surgery under conditions of insufficient muscle relaxation and under time constraints. A few days after surgery, the patient apparently suffered from edema and severe ascites with bile leakage; however, other than this, the patient showed no signs of liver failure or infectious complications.

In the 1950s, Lin (Taiwan) developed the so‐called "finger fracture method" in which the fingertips are used to crush the liver parenchyma in order to expose vessels needing to be ligated and divided. They reported having performed 48 cases of right hepatectomies and 34 cases of left hepatectomies using this method. Surgery‐related mortality within 1 month was 12.1%, and the 5‐year survival rate was 19%; outcomes which were considered excellent for that era.^5^

## 3 PREHISTORY OF LIVER ANATOMY

In Ancient Babylonia (2000 BC), fortune tellers determined people's fate on the basis of the shape of the liver of a sacrificed animal, and as shown by the sheep liver models at the British Museum, research on the morphology of the liver progressed as part of religious activities. Much later, in 1654, Glisson examined cast specimens of the liver in detail and published a book, Anatomia hepatis. The book was extremely well‐known for showing the basic anatomy of the liver's (triple) vascular system. Later, the method for the collective isolation of Glisson's capsule,^6^ developed by Takasaki et al, became the basic procedure for hepatectomy in many centers in Japan.

Up to the end of the 19th century, the segmental anatomy of the liver typically consisted of dividing the liver into “classical” right and left lobes along the falciform ligament, but because of the branched morphology of the portal vein and the presence of the avascular plane, a new concept was proposed by Rex (1888) and Cantlie (1897) in which the right and left lobes were divided by the line connecting the gallbladder bed to the inferior vena cava.^7^ This greatly influenced researchers in the 1950s, such as Hjorstjo, Healey, and Couinaud, who constructed the current concept of the segmental anatomy of the liver. In 1954, Couinaud used 120 cast models of the inner structure of the liver to explore the fissures, pedicles, and basic surgical segments of the liver by referring to the segments of the lungs and the segmentation of the liver in other animals. He then confirmed his findings through the dissection of 50 fresh organs and declared that the human liver could be classified into eight segments. Further, he proposed the concept of the limits of liver segmentation (fissures) and revealed that there was no intercommunication between the pedicles (blood vessels and bile ducts) of different segments. Later, in the 1980s, this concept became the basis of systematic subsegmental hepatectomy, which was developed by Makuuchi et al at the National Cancer Center.^8^

As for the definition of S1, Couinaud simply named the Spiegelian lobe "S1" and did not mention the concept of the paracaval portion (of the caudate lobe of the liver), proposed by Kumon. In 1985, Kumon examined the detailed anatomy of 23 cast specimens of human livers and showed that S1 was composed of three elements, namely, the so‐called Spiegelian lobe, the paracaval portion, and the caudate process, which in many cases have their own independent branches.^9^ Identifying the concept of the paracaval portion had a particularly powerful impact on liver surgery and has allowed the establishment of the anatomical definitions necessary to surgically address hilar cholangiocarcinoma^10^ as well as total caudate lobe resection for HCC.^11^

## 4 HISTORY OF HEPATECTOMY IN JAPAN AND ESTABLISHMENT OF THE LIVER CANCER STUDY GROUP OF JAPAN

The first hepatectomy in Japan was performed in 1929 by Ohno, who resected a primary liver cancer the size of a chicken egg. Later, in 1941, Ishiyama reported having performed a subtotal resection of the left hepatic lobe, and, in 1942, Muto reported having performed an atypical resection of two‐thirds of the right hepatic lobe through only ligature en masse of the hepatic parenchyma, basically stopping at the stage of atypical hepatectomy without dissection of the hepatic hilum. This led to the world's first typical right hepatic lobectomy, which, as mentioned earlier, was performed by Honjo in 1949.

The Liver Cancer Study Group of Japan was established in 1967, and in 1969 it started conducting nationwide follow‐up surveys of liver cancer patients in Japan. Thus far, the survey has been carried out once every 2 years, registering approximately 10 000 new cases per year and has led to the establishment of a valuable patient registry that is likely to be the largest in the world.

**Figure 1:**
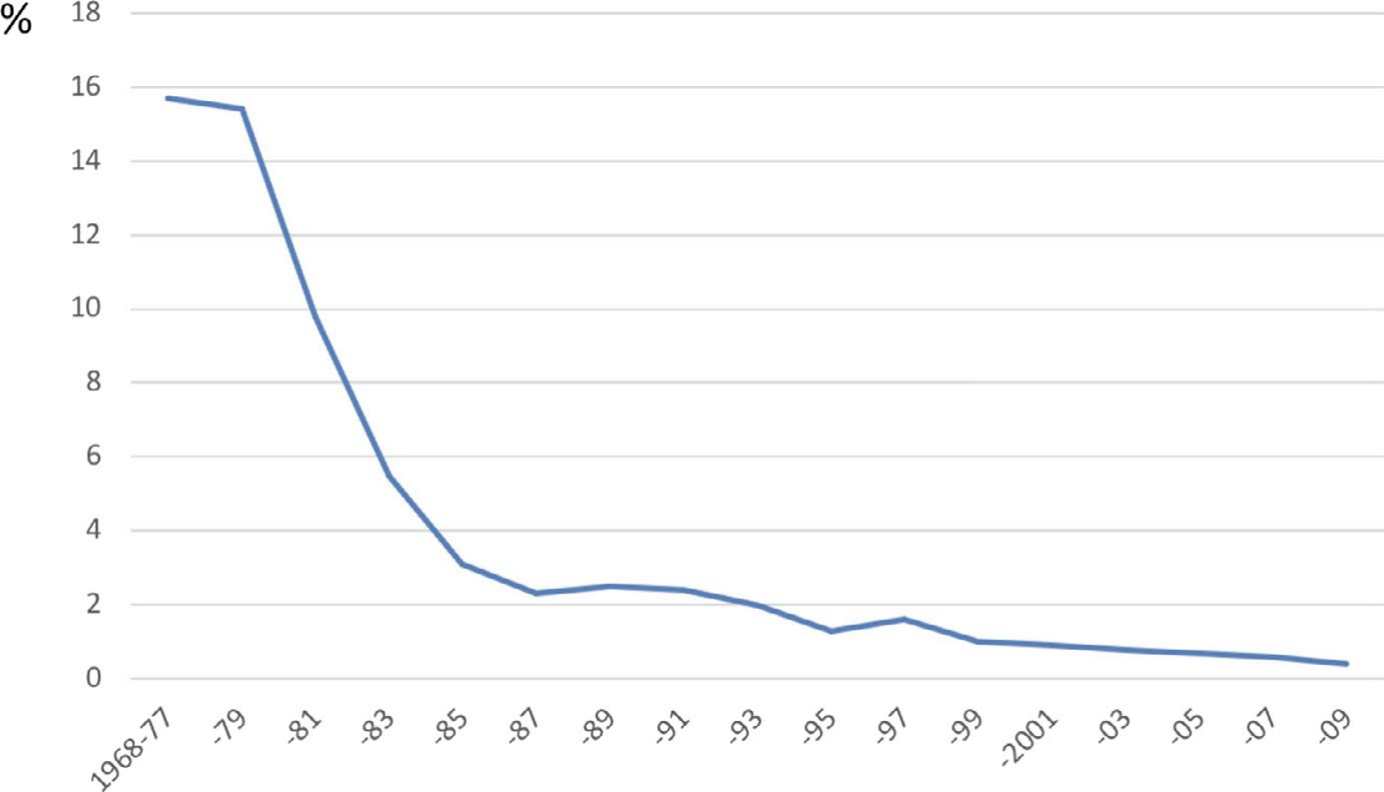
Variations in hepatectomy‐related mortality, according to data from a nationwide follow‐up study conducted by the Liver Cancer Study Group of Japan. In Japan, surgery‐related mortality after liver cancer resection exceeded 15% in the 1970s, but decreased rapidly in the 1980s and reached a level as low as approximately 1% in the late 1990s^13^

According to the initial report published by Okuda et al, a total of 360 liver resections for liver cancer were performed in 155 facilities nationwide during the 10‐year period from 1968‐1977.^12^ Their calculations showed that there were as few as 2.3 cases per facility per 10 years, and when the total number of cases (4031) was used as the denominator, the liver resection rate was only 9.0%. By comparison, the number of patients subjected to exploratory laparotomy during that same period was 518, which is indicative of the difficulty of obtaining a preoperative diagnosis. In 222 cases of resection due to HCC, the mortality rate within 1 month of surgery was as high as 27.4%, and the 3‐year and 5‐year survival rates were as poor as 19.6% and 11.8%, respectively. According to the aggregated data published by Arii, surgery‐related mortality after liver cancer resection in Japan exceeded 15% in the 1970s, but decreased rapidly in the 1980s and reached a level as low as approximately 1% in the 1990s^13^ (Figure 1).

## 5 1980S‐1990S: THE ERA OF MODERN LIVER SURGERY PIONEERS

**Figure 2:**
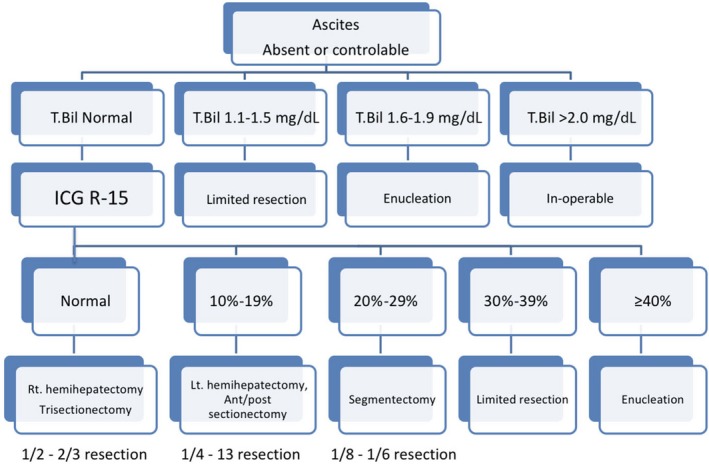
The Makuuchi Criteria for the safety limit of liver resection (cited from Ref. 14 with modification)

While several factors may have led to the rapid decrease in mortality in the 1980s, the establishment of safety limits for the extent of liver resection in injured livers played a major role. In Japan, when determining resectability, the severity of liver impairment is accurately assessed on the basis of the indocyanine green (ICG) tolerance test. The so‐called "Makuuchi criteria" for hepatic resection, which include ICG clearance, are widely used in Japan and other Asian countries^14^ (Figure 2). The data accumulated using these criteria accounts for several hundred patients who underwent liver resection at the National Cancer Center until the 1980s, and although the criteria were developed empirically rather than using a formal statistical method, it has greatly contributed to the safety of liver resection. Other liver function tests using asialo‐scintigraphy,^15^ biliary scintigraphy,^16^ and gadoxetic acid (Gd‐EOB‐DTPA) enhanced magnetic resonance imaging (EOB‐MRI)^17^ are currently available and used complementarily and sometimes as tools for the measurement of segmental liver function.

As for technological advances, intraoperative ultrasonography has had a great impact on the safety and quality of liver resections. Intraoperative ultrasonography has made it possible to visualize, in real time, the vascular anatomy of the portal and hepatic veins, which pass through the liver in a complex layout. Makuuchi et al quickly adopted the use of intraoperative ultrasonography and developed a systematic subsegmental resection, which ensured a balance between oncological curability and safety.^18^ To carry out a systematic subsegmental resection, surgeons need to identify the areas perfused by the portal vein in the cancer‐bearing subsegment; as such, an ultrasound‐guided dye injection method (for indigoid dyes) was simultaneously reported. Although no reported randomized controlled study has shown the superiority of systematic liver resections for HCC, its usefulness has been demonstrated by a number of retrospective studies.^19^, ^20^

**Figure 3:**
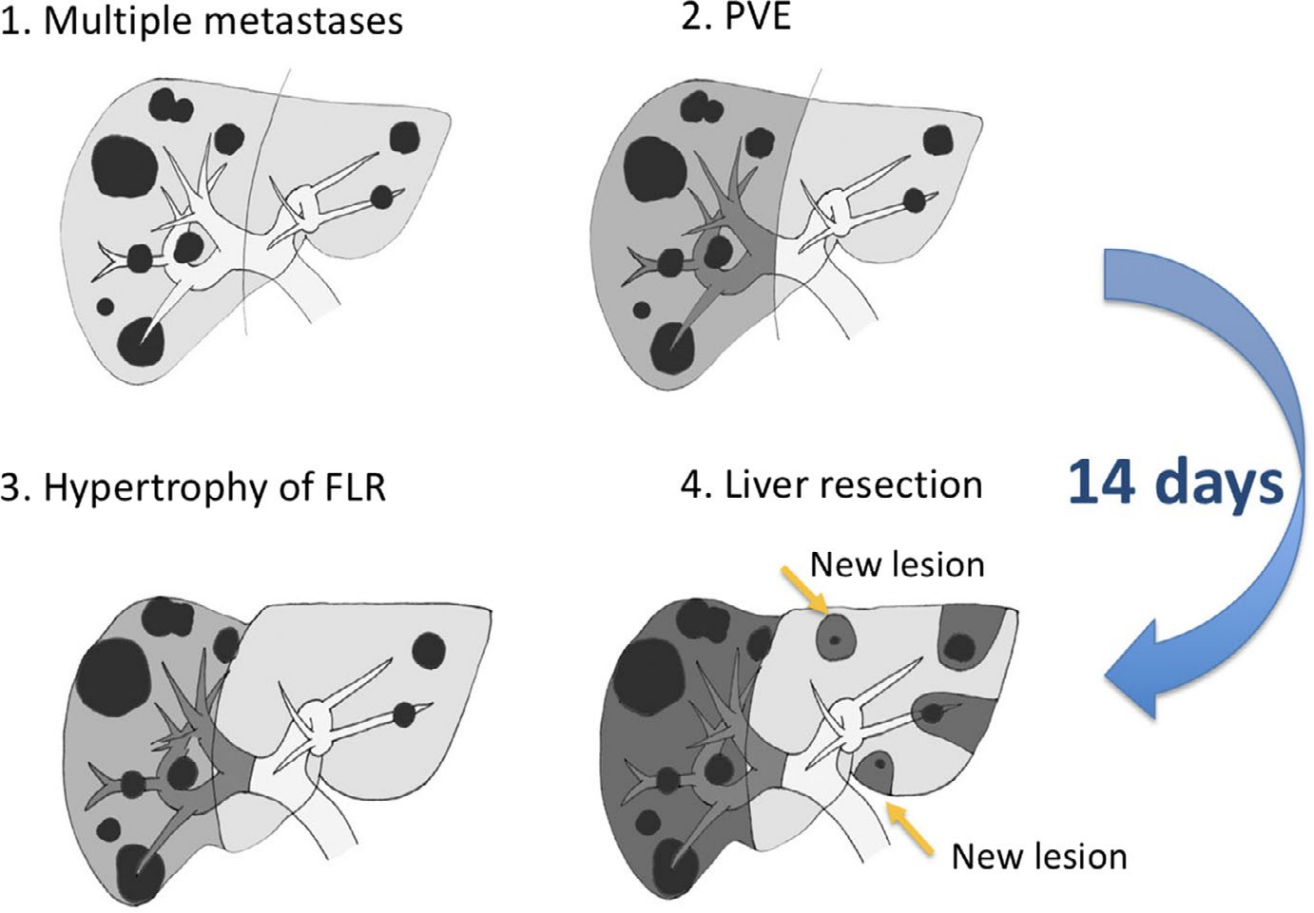
Safe hepatic lobectomy using portal vein embolization for the treatment of multiple liver metastases

Portal vein embolization for the prevention of liver failure, after extended resection, was also developed in the 1980s.^21^ In a patient with a hilar cholangiocarcinoma obstructing the right branch of the portal vein, Makuuchi et al noted a phenomenon in which the left lobe of the liver was enlarged in order to functionally compensate. Thus, they developed a method consisting of artificial embolization of the right branch of the portal vein in order to cause atrophy of the resected side and enlargement of the remaining liver. Introduction of this method has markedly improved the safety of the extended lobectomy in the treatment of hilar cholangiocarcinoma and has since been applied to the surgical treatment of multiple liver metastases^22^ and HCC^23^ (Figure 3).

## 6 PROGRESS SINCE 2000 [1]: LAPAROSCOPIC HEPATECTOMY

The major drawback of open liver resection is the large laparotomy wound. Laparoscopic surgery, which developed rapidly in the 1990s, has gradually been used to perform hepatectomies. Laparoscopic hepatectomy was first reported by Reich in 1991,^24^ and, in Japan, Kaneko^25^ began using the technique in 1993. Since then, its use has gradually been accepted. The Endoscopic Liver Surgery Study Group was established in 2006, and the cases of laparoscopic hepatectomy in Japan have been registered annually. In the initial report published by Tsuchiya et al, 471 cases from 26 facilities were counted, but most consisted of either a resection of the lateral section of the left liver (left lateral sectionectomy) or a wedge liver resection, and pure laparoscopic hepatectomy surgery accounted for only 47% of cases.^26^ In 2010, lateral sectionectomy and wedge liver resection were approved for social insurance coverage, and since then, have been attempted in many centers in Japan. According to the fourth report, which was released in 2011, the number of hospitals performing these procedures increased to 113, and the number of patients receiving laparoscopic liver resections increased to 2899. The number of patients treated using pure laparoscopic surgery, similarly, increased to 63%. It was anticipated that highly challenging liver resections, such as the hepatic lobectomy, would also be covered by health insurance, but issues arose due to multiple deaths following laparoscopic hepatectomy and pancreatectomy in some university hospitals. In 2016, highly challenging surgical procedures, such as subsegmentectomy and more extensive operations, were finally approved for insurance coverage under the condition that all patients would be registered prospectively. Since late 2017, 4095 patients have been registered, including 891 cases of subsegmentectomy and other more extensive procedures, and the mortality rate within 90 days has reportedly been 0.67%, which is much lower than that found in Western countries. However, according to the latest International Consensus Meeting, laparoscopic hepatic lobectomy is still considered an exploratory procedure and is not yet performed in routine surgical care, as it is a surgical procedure that requires significant expertise and has a steep learning curve.^27^ Expectations are high regarding the application of minimally invasive robotic surgery to the field of hepatic surgery. However, due to issues such as expensive medical costs, limitations in the adjustment to patient posture or position, as well as differing opinions regarding the suitability of laparoscopic surgery for large organs like the liver, the procedure has only been attempted in a limited number of facilities. Nonetheless, the latest reports have shown that robotic surgery is better suited for performing the right hepatectomy than laparoscopic surgery in terms of surgery time (duration of surgery) and the rate of conversion to laparotomy;^28^ thus, future progress is expected.

## 7 PROGRESS SINCE 2000 [2]: 3‐DIMENSIONAL SIMULATION AND NAVIGATION TECHNOLOGY

**Figure 4:**
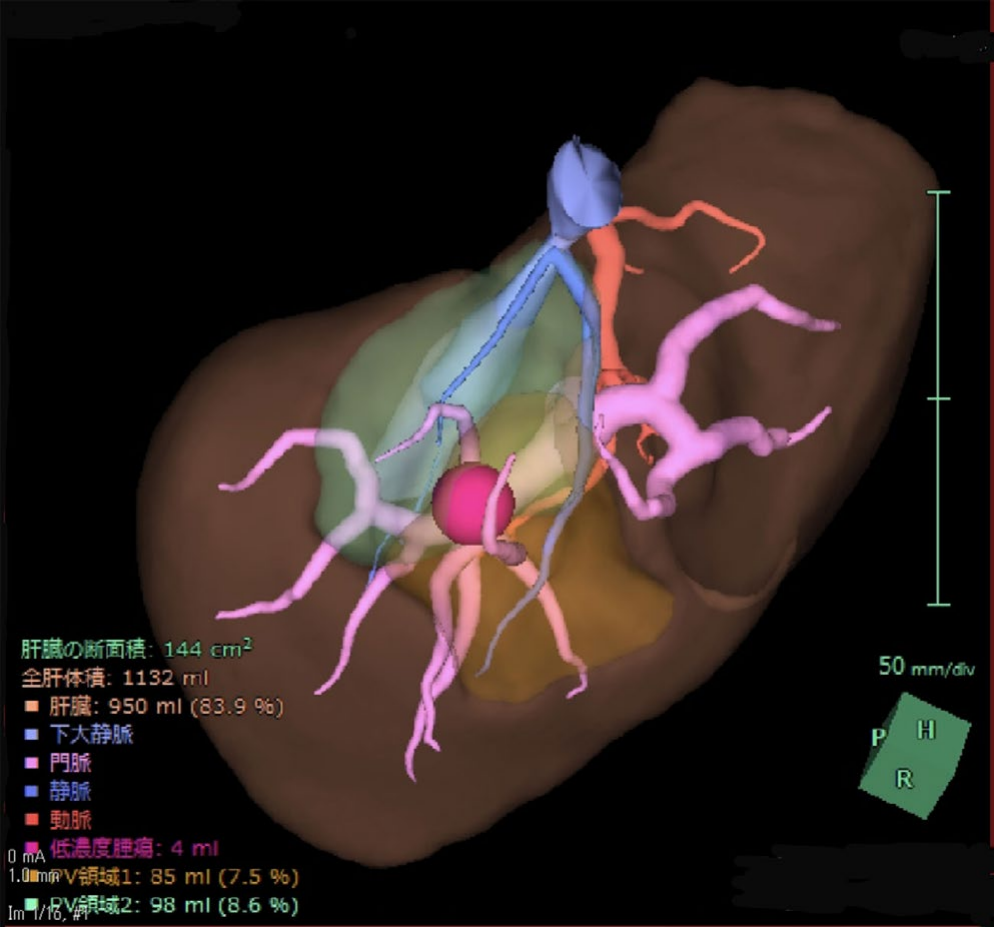
Typical case of liver navigation. In this patient, the treatment plan consisted of anatomic resection of the dorsal and ventral parts of S8 for the treatment of a small HCC

Three‐dimensional (3D) simulation technology differs from conventional 3D image display as it allows for the calculation of the volume of the areas perfused by intrahepatic blood vessels. Three‐dimensional simulation technology was developed in Germany in the early 2000s,^29^ and immediately thereafter software based on an original algorithm was developed in Japan. This software has been widely used to assess the indicators for reconstruction of the middle hepatic vein branches in right liver grafts during living‐donor liver transplantations.^30^ In 2008, the technology was recognized as part of advanced medicine and has since been applied to complex hepatectomies involving hepatic vein reconstruction as well as systematic subsegmental hepatectomies^31^ (Figure 4). Its usefulness has been demonstrated in facilities specializing in liver surgery all over Japan, and, since 2012, it has been approved for insurance coverage. An analysis conducted by Mise et al, on more than 1000 cases of 3D simulation, has shown that the technology is essential for the determination of the appropriate surgical procedure to be performed on the donor in living‐donor liver transplantations. Likewise, in the treatment of HCC, the number of cases in which systematic resection is indicated has increased, and findings have shown that 3D simulation may be excellent from an oncological perspective as well. In patients with multiple liver metastases, this has led to an increase in the number of patients undergoing surgery aimed at achieving R0 resection (complete resection) through complex procedures.^31^

The next step after 3D simulation technology is advanced navigation technology that provides real‐time intraoperative guidance during liver transection. Technology is currently being developed at many facilities in pursuit of this aim.^32^ However, due to certain properties of the liver, such as liver plasticity and changes in liver position during surgery, there is still a large spatial navigation error of approximately 10 millimeters,^33^ and many issues still need to be resolved, including a reduction in the time required for position adjustment.

## 8 PROGRESS SINCE 2000 [3]: ADVANCES IN INTRAOPERATIVE IMAGING

As mentioned above, significant progress was made due to intraoperative ultrasonography in the 1980s, and it has been widely used ever since. In the 1990s, the first‐generation ultrasound contrast agents were released on the market, but were not widely used because there was no identified advantage to their application in intraoperative ultrasonography. In 2009, the second‐generation contrast agent Sonazoid (perflubutane) displayed excellent results in the evaluation of tumor blood flow in the early phase of contrast enhancement and provided higher‐definition images of tumors in the Kupffer phase of the delayed phase of contrast enhancement; thus, it rapidly became popular. In HCCs, as well as in metastatic liver cancers, intraoperative ultrasonography using Sonazoid has reportedly allowed for the detection of new lesions that were not identified preoperatively,^34^, ^35^ and, as a result, the procedure has become an essential modality in modern liver surgery.

**Figure 5:**
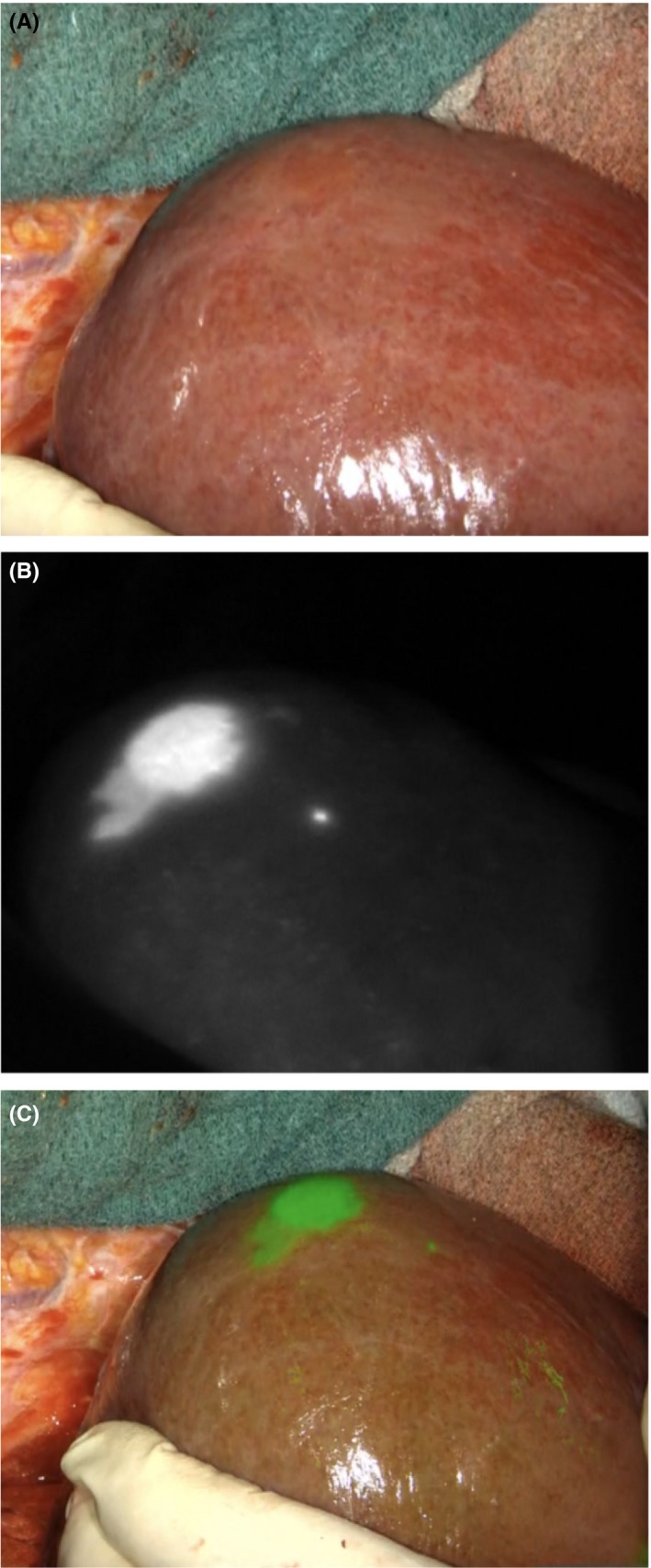
Typical case of ICG‐positive hepatocellular carcinoma. The fluorescence camera (B: black‐and‐white mode, C: green overlay mode) reveals the presence of a small HCC lesion directly under the surface of the liver, which is impossible to recognize with the naked eye (A)

**Figure 6:**
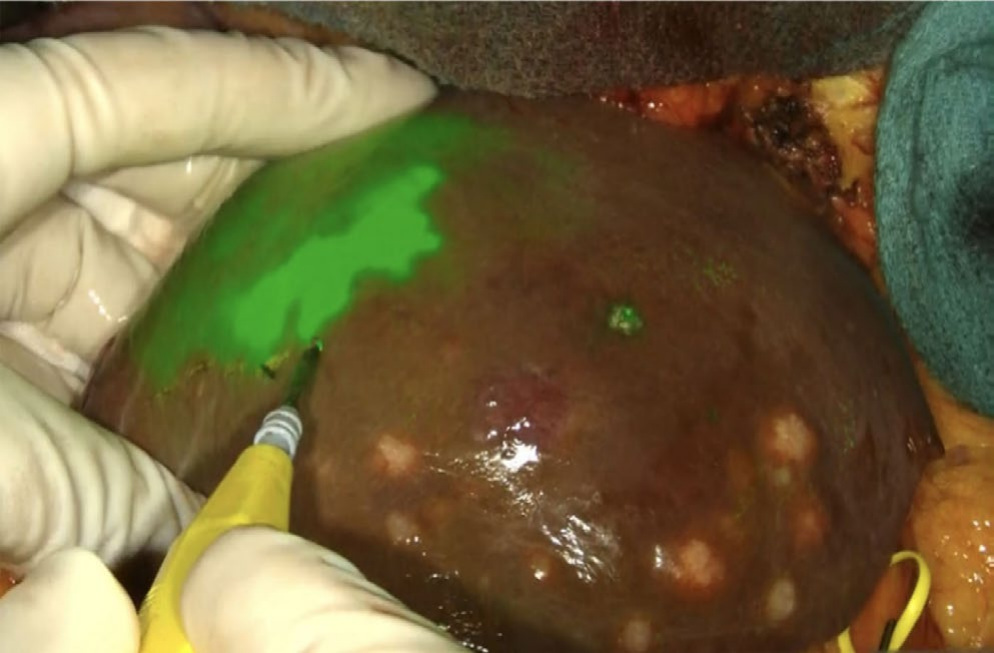
ICG staining of hepatic segments. In this case, trace amounts of ICG is injected intraoperatively with ultrasound guidance into the portal vein branch, which perfuses the hepatic segment next to the one containing the tumor. The stained area is displayed with green fluorescence, and the boundaries between the hepatic segments are clearly visible

Around the year 2007, ICG fluorescence imaging was first introduced as a completely new imaging technology. ICG is a green dye, which, for decades, has been used for the evaluation of liver function. When trace amounts of ICG are mixed with blood or bile and the mixture is excited with 750‐810 nm near‐infrared light, near‐infrared fluorescence of approximately 840 nm is emitted. A camera designed to visualize near‐infrared fluorescence was developed and, as a result, the procedure has been used for cholangiography^36^, ^37^ and for the evaluation of organ blood perfusion. It was found that HCCs that nest themselves became fluorescent, and findings revealed that ICG administered preoperatively for the purpose of conducting liver function tests was also absorbed by the cancer nests and retained there for some reason^38^, ^39^ (Figure 5). In cases of metastatic liver cancer, findings have shown that ICG is not absorbed by the tumor itself, but is instead accumulated in a ring‐shaped manner in the liver parenchyma around the metastatic lesion and produces fluorescence.^38^, ^40^ Meanwhile, in systematic liver resections, Aoki et al reported that when trace amounts of ICG are introduced by puncturing and staining the branches of the portal vein, the boundaries between the hepatic segments become clearly visible in a reproducible manner^41^, ^42^ (Figure 6). In contrast, when ICG is administered systemically following the interruption of blood flow to the side that is scheduled to be resected, the entire liver becomes fluorescent, while the area with interrupted blood flow stands out because of the absence of fluorescence. This is known as the negative staining method and has also been widely used.^42^, ^43^

## 9 PROGRESS SINCE 2000 [4]: BROADENING OF THE INDICATIONS FOR HEPATECTOMY (ALPPS)

As an additional measure to broaden the indications for hepatectomy, a new two‐stage hepatectomy procedure was proposed in 2012.^44^ In this procedure, only ligation of the right branch of the portal vein and transection of the liver are performed during the first surgery, and, approximately 9 days later, an extended right hepatectomy is performed during a second surgical procedure. This method was named "associating liver partition and portal vein ligation for staged hepatectomy" (ALPPS) by Santibanez et al.^45^ The procedure drew attention because of the accelerated speed of regeneration of the predicted residual liver, and because there were fewer dropouts during the waiting period. However, a notable disadvantage is the surgery‐related mortality rate of 20% (or higher). Since the early development of this method, an international registry has been established,^46^ and the procedure has been performed experimentally in many facilities worldwide. With transparency ensured, the number of registered cases has already exceeded 1100. Improvements have also been made with regard to partial hepatic transection, which already had a low mortality risk, and surgical safety has gradually improved.^47^, ^48^ Despite this, there have been recent questions regarding the oncological benefits of ALPPS. Olthof et al compared the long‐term outcomes of ALPPS with those of systemic chemotherapy in the treatment of advanced colorectal cancers with multiple liver metastases and reported that there were no apparent differences between the two treatments.^49^ In order for ALPPS to remain a valid surgical procedure in the future, additional careful studies will need to be executed.

## 10 FIFTY YEARS OF LIVER TRANSPLANTATION

Liver transplantation is an extremely effective treatment for end‐stage liver failure. The world's first liver transplantation from a brain‐dead donor was performed by Starzl et al in 1963 on a 3‐year‐old pediatric patient with congenital biliary atresia in Denver, Colorado (USA); however, the patient died intraoperatively due to massive bleeding. Later, liver transplantation was carried out in four other cases in the same facility, as well as in Boston and Paris (one case each); but, in all cases, the patients died shortly after surgery due to postoperative complications.^50^ Thus, because the initial outcomes were poor, there was a global moratorium, and institutions voluntarily refrained from performing liver transplantations for 3.5 years, from early 1964 until the middle of 1967. Finally, with developments in surgical technique and the development of immunosuppressive drugs, survival for more than 1 year was achieved for the first time in 1967.

Since the 1980s, with the availability of effective immunosuppressants such as cyclosporine and tacrolimus, the outcomes of liver transplantation have improved dramatically, and the number of transplant cases has also increased substantially. Nowadays, in the leading industrialized countries in Europe as well as in the USA, liver transplantation from brain‐dead donors has become an extension of routine medical care in the treatment of liver disease. The 1‐year survival rate has been higher than 80%, and the 5‐year survival rate has been higher than 70%.

The world's first living‐donor liver transplantation in children was attempted in 1988 in Brazil, and the first successful case was reported in 1989 in Australia, where transplantation was performed between a Japanese mother and her child.^51^ In the same year, in Japan, living‐donor liver transplantation in children was attempted by Nagasue et al at Shimane Medical University. In 1993, Makuuchi et al from Shinshu University successfully performed the first procedure in the world in adult patients.^52^ This success showed that living‐donor liver transplantation could be considered a possible alternative to whole‐liver transplantation from brain‐dead donors, which was the only treatment option at the time for patients with liver failure in countries like Japan, where liver transplantation from brain‐dead donors was almost impossible.

The first liver transplantation from a brain‐dead donor in Japan was performed by Kawasaki et al from Shinshu University in 1999 after the "laws on organ transplantation" were enacted in 1997. Initially, the donors themselves, while still alive, were required to express their willingness to donate their liver after death, and for this reason there were less than 10 cases per year in all of Japan. However, in 2009, the law was revised, and organ donations are now allowed upon consent from the donor's family. As a result, the number of cases has increased to more than 50 per year. According to the aggregated data published by the Japan Organ Transplant Network, the number of donations increased to 79 in 2017, bringing the total number to 499. However, even under these conditions, approximately 400 living‐donor liver transplantations are currently needed every year, and the Japanese Society for Transplantation and the Japan Organ Transplant Network are working on increasing awareness.

The so‐called "Milan criteria,"^53^ which were proposed in 1994, have thus far been the gold standard for the applicability of liver transplantation in the treatment of HCC. Previous research papers suggested that the indications be broadened, and many additional criteria have been proposed. However, none have become widely used, with the exception of the University of California San Francisco (UCSF) criteria.^54^ There were calls for the creation of Japanese criteria to broaden the indications for liver transplantation, most of which referred to living‐donor liver transplantations, and recently, the 5‐5‐500 criteria were announced.^55^ These criteria were statistically determined based on data from more than 1000 nationwide cases of living‐donor liver transplantations for patients with liver cancer, including individuals that fulfilled certain conditions (maximum tumor size of 5 cm, a 70% or higher 5‐year survival rate, and a 10% or lower 5‐year incidence of recurrence), and were also based on the optimal criteria for alpha‐fetoprotein (AFP) or protein induced by vitamin K absence or antagonist‐II (PIVKA‐II). These criteria, which require five or fewer tumors of 5 cm or less, as well as AFP levels of 500 ng/mL or lower (no extrahepatic metastasis, no vascular invasion), are already being used as the Japanese criteria for registration for liver transplantation from brain‐dead donors and may potentially be used among other criteria for insurance coverage of living‐donor liver transplantation.

## 11 CONCLUSIONS

Tremendous progress in the field of liver surgery has been made over the past 50 years. The widespread use of hepatectomy and the improvement in outcomes have mainly been due to technological advances achieved by pioneers in the last century, as well as rapid improvements in safety. Whole‐liver transplantation from brain‐dead donors began being performed in the 1960s; however, acceptable outcomes were only achieved in the 1980s as a result of the development of novel immunosuppressants and technological progress. Partial liver transplantations from living donors, which began being performed in the 1990s, required further improvements in advanced technology and perioperative management, and technological development in that regard has contributed to overall improvements in the entire field of liver surgery. Since the turn of the century, safer and more extensive detailed surgeries have been made possible due to advances in computer and imaging technology. Laparoscopic surgery and robotic surgery have solved the issue of large incisional wounds, a major drawback of open liver surgery, but further innovation will be needed in order for safety and accuracy that is comparable to open abdominal surgery to be achieved in all surgical procedures. Liver surgery in the near future will be more precise and less invasive, supported by substantial progress in technologies surrounding liver surgery, including navigation surgery, cancer imaging, and minimally invasive surgery. This overview of the history of liver surgery over the last 50 years may provide useful insights for further innovation in the next 50 years.

## DISCLOSURE

Authors declare no conflict of interests for this article.
